# Case of Obscure Disease of the Brain

**Published:** 1862-06

**Authors:** 


### ART. IV.—*Case of Obscure Disease of the Brain.*

[The following is a private letter which we have received, and which, from its exceeding interest, we desire to publish. We trust our friend will excuse the liberty we have taken.— [Ed.

Dr. Miner—Dear Sir:

I desire to lay before you a concise history of my little girl, now four years nine months old, and hope you will give it a careful perusal, and recommend any treatment you can in your better knowledge and judgment of disease than the anxious parent, of the little one, of which he speaks. She was taken ill on the 23d of August, 1860, being then two years and ten and a half months of age. Had a good constitution, and was generally healthy, and perhaps more intellectual than most children. Could repeat the Lord's Prayer readily; could pronounce every letter of the alphabet distinctly; could sing several childish songs, and had a most accurate ear for music.

On the day of which I speak, (it being very warm,) she had been playing about the garden, protected by a sun-bonnet, till about six o'clock in the afternoon, when she came in and told her mother that her head ached. (She also had eaten pretty freely of green corn for dinner, and some apple pudding.) Mrs. C. laid her down upon a sofa, and she slept for an hour, and then awoke with a most violent fever. I was away from home at the time; on being sent for, I arrived at half past eight, just in time to see her go into most violent convulsions, which lasted for three hours and a half, though we brought every means in our power to bear upon the case. Toward morning she had strong symptoms of another, and I may say was stupified and convulsed more or less all night. The following day I gave her castor oil and laudanum, kept warm stupes to the abdomen, as she had pains about every twenty minutes similar to a woman in the first stages of labor. On Saturday afternoon she had another, which lasted one-half or three-fourths of an hour. "*Alta so sick*," the last words she spoke for about five months.

On Sunday no better; the pains spoken of still keeping up, in spite of all the warmth and anodynes which we could give or apply. On Sunday night about 12 o'clock, despite everything my brother-in-law and myself could do, she had spasms again, and we had resort to almost every remedy except chloroform and sulphuric ether. I kept her under the influence of calomel and opium, and on Monday evening gave her Castor oil and an injection, and then for the first time did she have a free dejection, such as I desired to see; for though we attributed the attack to a sun-stroke, yet I felt certain that the corn had something to do with those pains, for she passed a large quantity of undigested hulls.

I said above, she went into spasms on Sunday night, and they continued upon her without intermission till Tuesday morning; but dating from the first attack they commenced Thursday night, and lasted till Tuesday morning, a period of four days and five nights. Why I am so pointed is to show you the time the brain suffered, when both patient and convulsions seemed worn out and exhausted. And still there were present from time to time fugitive pains in the bowels; pressure with the hand over the warm stupes always gave relief, and there never was the least tympanites but the abdomen was soft and relaxed. From that time onward she has been gradually recovering in strength and intellect. For three months, she was so nervous that if standing upon the floor and the door opened, she would cry out and fall down. She improved of that, and at the present time (April 9, 1862,) will say five or six words connected, or in singing will connect a dozen, as she catches a tune very easy. But that which alarms me is, about a year since I observed that she would take little spells that drew her head down, and left a shudder and increased respiration, which would last about one-half of a minute, and as soon as it passed away would run about again as usual. Lately I think they are harder, but would not exceed much the above time. I think the pupil of the eye always dilates, and the pulse is increased, and the face flushed at each attack, and she moans so hard sometimes that you can hear her if you are up stairs. She never falls, but sometimes turns partly around. If she has hold of her doll, or any plaything, she does not let it go. Never froths or foams at the mouth; never bites or protrudes her tongue in any attack; the number varies from three to six daily; never any in her sleep, (I always sleep with her;) sleeps well; has more in the house than when out with the girl, or out driving with me. There is a tendency to restlessness, and playing with things for a short time. She is passionately fond of books and pictures. There is no hereditary disease in either family. She grows as well as any child. I fear that epilepsy is about to follow an excited state of the nervous system. Perhaps the brain and its membranes may have suffered from heat of the sun's rays, though the whole attack pointed to the bowels at the time.

Dr. Miner, I am at times disheartened, for what Mrs. C. and I have passed through in mind and body, the sleepless nights, the anxiety for nearly two years, a Higher Power only knows; an only child, the pride of our house, to be thus prostrated, and the parents kept in doubt, in fear, and yet at times, hope. Pardon the liberty I have taken with you. Please give us your advice and you will be thankfully remembered.

Yours, truly, G. C.

P. S.—I may mention that those spells have drawn the right eye or turned it a little inward. It shows more if she has the spells often.

